# 探索microRNAs在人类癌症中的治疗潜能

**DOI:** 10.3779/j.issn.1009-3419.2012.08.10

**Published:** 2012-08-20

**Authors:** William CS CHO, 燕 丁

**Affiliations:** 1 Department of Clinical Oncology, Queen Elizabeth Hospital, Hong Kong; 2 天津医科大学总医院，天津市肺癌研究所，天津市肺癌转移与肿瘤微环境重点实验室; 3 香港特别行政区 伊利沙伯医院 临床肿瘤科

**Keywords:** 癌症, microRNA, miRNA, 肿瘤miRNA, 治疗靶标

## Abstract

大量研究表明microRNAs（miRNAs）的异常调节与癌症的发生和进展相关。新近研究发现了若干在各种人类癌症中具有可作为治疗靶标巨大潜能的miRNAs。这些肿瘤miRNAs的抑制或过表达可调节相关基因的表达，从而抑制各种癌症的增殖或转移。一些miRNAs可逆转上皮-间质转化的表型，有些则可用于增强细胞对抗癌药物的敏感性。它们大部分的抗癌作用均已在临床前动物模型中得到验证。miRNA治疗的一个优点是它可靶向作用于不同信号通路中的许多基因，但同时亦伴有许多未知的脱靶效应的缺点。此外，对于有效的miRNA治疗来说，成功转运也是一个主要的挑战。然而，新近研究的发现及药物转运系统的高速发展为该领域的飞跃展现了一个乐观的前景。

## 1 前言

大量研究表明microRNAs(miRNAs)的异常调节与癌症的发生和进展相关。研究发现许多miRNAs可作为致癌基因、抑癌基因、以及肿瘤干细胞和转移的调节剂^[1]^。随着对miRNA靶基因及其对细胞影响的深入了解, 调节miRNA的表达或可为癌症治疗提供令人振奋的机会^[2]^。

## 2 miRNAs作为潜在的治疗靶标和工具(表 1)

**1:** 文中各种类型癌症中的miRNAs及其靶基因

miRNAs	在癌症中的调节异常	靶基因	分子机制	参考文献
let-7	在肝细胞CSCs中增加	SOCS-1、CASP3	抑制let-7可增加肝细胞CSCs对多柔比星和索拉非尼的化疗敏感性	[5]
miR-10b	在转移性乳腺癌中增加	CCN5	经由CCN5通过TWIST1的表达抑制而抑制：该抑制由HIF-1*a*的转化抑制与修饰通过阻碍JNK信号通路而介导	[14]
miR-15b, miR-200b	在化疗耐药的舌癌中降低	BMI1	有效逆转EMT的表型并使癌细胞对化疗敏感	[24]
miR-24-2	在乳腺癌中降低	BCL-2	通过调节不同的凋亡通路而诱导凋亡	[17]
miR-34b/c	在甲基化MPM细胞中降低	c-MET	具有抗增殖作用，使细胞发生G1期细胞停滞，并可抑制癌转移、侵袭及活跃性	[11]
miR-101	在胰腺的IPMN及PCa中降低	EZH2、COX-2	通过上调EZH2, miR-101的缺失可启动IPMN的腺癌序列。离体及在体试验中，外源性miR-10可抑制离体和在体PCa细胞的增殖和生长	[7, 8]
miR-125b	在HCC中降低	PIGF	降低细胞增殖、非停泊性生长、细胞转移、侵袭及血管新生	[12]
miR-148a	在GC中降低	ROCK1	抑制在离体试验中GC细胞迁移以及在体试验中肺癌转移灶的形成	[21]
miR-155	在BRCA1缺失肿瘤中增加	BRCA-1	BRCA1通过与HDAC2联合，使miR-155启动子中的组蛋白H2A和H3去乙酰化，从而在表观遗传学水平抑制miR-155的表达	[3]
miR-181	在肝细胞CSCs中增加	RASSF1A、TIMP3、NLK	miR-181沉默可导致肝细胞CSCs活跃性及侵袭性的减弱	[5]
miR-193a	在p53缺失肿瘤中增加	p53	化疗可诱导p63/p73依赖性miR-193a-5p, 并通过miRNA介导的p73的反馈性抑制而限制化疗敏感性	[4]
miR-199a/b	在HCC中降低	PAK4	通过抑制PAK4/Raf/MEK/ERK通路而抑制离体和在体HCC的生长	[15]
miR-200a	在胰腺癌中降低	DCAMKL-1	在癌细胞中si-RNA介导的DCAMKL-1的敲除可诱导miR-200a及EMT转录因子ZEB1、EZB2、Snail、Slug和Twist的下调	[23]
miR-340	在侵袭性乳腺癌中降低	c-Met	通过调节MMP-2及MMP-9的表达抑制肿瘤细胞转移和侵袭	[19]
miR-342	在CRC中降低	DNMT1	明显降低DNMT1的表达，从而通过启动子去甲基化重新激活ADAM23、Hint1、RASSF1A及RECK基因	[10]
miR-488*	在PCa中降低	AR	下调AR的转录活性并抑制外源性AR蛋白的生成	[16]
miR-499	在高侵袭性CRC中增加	FOXO4、PDCD4	促进离体CRC细胞的转移和侵袭以及在体肺和肝转移	[18]
miR-516a	在高转移性GC中降低	Sulfatase 1	端胶原介导miR-516a-3p表达载体传送至原位高转移性肿瘤中，在阻断GCs的转移扩散中具有应用价值	[22]
miR-520b	在高转移性乳腺癌中降低	HBXIP	HBXIP刺激NF-sB介导的IL-8表达促进癌细胞的转移网络来调节癌细胞转移	[20]
miR-558	在神经母细胞瘤中通过MYCN而抑制	宿主基因BIRC6	明显增加在体的肿块形成、增殖和肿瘤生长	[6]
miR-591	在神经母细胞瘤中通过MYCN而抑制	宿主基因SLC25A13	在原位神经母细胞瘤异种移植动物中具有很强的肿瘤抑制效应	[6]
miR-708	在RCC中降低	ZEB2、BMI1	降低细胞生长、可克隆性、侵袭及转移，并明显增加细胞凋亡	[9]
AR:雄激素受体；CRC:结直肠癌；CSCs:肿瘤干细胞；EMT:上皮-间质转化；GC:胃癌；HCC:肝细胞癌；IPMN:管内乳头状粘液肿瘤；miRNA: microRNA; MPM:恶性胸膜间皮瘤；PCa:前列腺癌；RCC:肾细胞癌。				

### 2.1 miRNAs在癌症发展中的作用

BRCA1是一个抑癌基因, 在受损DNA的修复和细胞周期调节中发挥作用。一项研究发现BRCA1联合HDAC2可使miR-155启动子中的组蛋白H2A和H3去乙酰化, 从而在表观遗传学水平上抑制miR-155的表达。miR-155的过表达可加速体内肿瘤细胞的生长, 而敲除miR-155则可减弱其生长。该研究结果提示miR-155是BRCA1缺失肿瘤的潜在治疗靶标^[3]^。p53是另一众所周知的抑癌基因, 然而p53相关的转录因子p63和p73在鳞癌中均过表达。一项研究发现化疗可引发p63/p73依赖性miR-193a-5p的诱导, 从而通过miRNA介导的p73的反馈性抑制以限制化疗敏感性。抑制miR-193a可中断该反馈, 抑制离体和在体肿瘤细胞的生存并增强化疗敏感性。因此在p53缺失肿瘤中, miR-193a的抑制或可提供新的治疗机会^[4]^。

肿瘤干细胞(cancer stem cells, CSCs)具有广泛的增殖和自我更新潜能以促进癌症的发展。有研究显示let-7和miR-181在肝细胞CSCs中表达上调。还表明let-7靶向作用于SOCS-1和CASP3, 而miR-181则靶向作用于RSSF1A、TIMP3及NLK。let-7的抑制可增加肝细胞CSCs对多柔比星和索拉非尼的化疗敏感性, 而miR-181沉默可减弱肝细胞CSCs的活跃性和侵袭性^[5]^。MYCN是神经母\细胞瘤癌变的主要驱动因子, 一项全基因组研究显示, 在高MYCN环境中MYCN靶标miRNA启动子受不同程度的抑制, miR-558明显增加在体的肿块形成、增殖和肿瘤生长, 而miR-591则在原位神经母细胞瘤异种移植动物中呈现很强的肿瘤抑制效应。该数据揭示了MYCN靶标miRNAs的非宿主基因依赖性功能, 并显示MYCN可同时抑制促增殖性及肿瘤抑制性miRNAs^[6]^。

为探索管内乳头状粘液肿瘤(intraductal papillarymucinous neoplasm, IPMN)的癌变机制, 一项研究显示与胰腺良性IPMN相比, 恶性IPMN中miR-101表达较低而EZH2表达则较高。miR-101在转录后靶向作用于EZH2, 通过上调EZH2, miR-101的缺失可启动IPMN的腺癌序列, 该研究提示miR-101-EZH2的阻断可作为IPMN癌变的潜在治疗靶标^[7]^。癌变与慢性炎症相关, 而在肿瘤组织中常伴COX-2的过表达。另一项研究发现COX-2是miR-101调节转录后的直接靶标, 外源性miR-101可抑制离体及在体前列腺癌(prostate cancer, PCa)细胞的增殖和生长。这些数据显示通过直接抑制COX-2的表达, 外源性miR-101或可提供一种新的癌症治疗方法^[8]^。

慢性应激可削弱个体的免疫系统, 这或可增加患癌风险。研究发现在肾细胞癌(renal cell carcinoma, RCC)中miR-708是一个抑癌基因, 在应激控制中发挥作用。在RCC细胞中miR-708表达的恢复可降低癌细胞的生长、可克隆性、侵袭及转移, 并可明显增加细胞凋亡。miR-708在肿瘤内的转运可引发异种移植动物内肿瘤的在体萎缩。E-cadherin调节剂ZEB2和BMI1已被确定为疑似miR-708靶标, 这些发现为RCC的治疗提供了新的靶标^[9]^。事实上, 在人类癌症中已发现许多癌基因, miRNA可在转录后调节这些癌基因的表达。在结直肠癌(colorectal cancer, CRC)中, DNMT1的过表达促进了抑癌基因的沉默。一项研究显示恢复已下调的miR-342可引起CRC细胞中DNMT1表达的明显下降, 从而通过启动子去甲基化重新激活ADAM23、Hint1、RASSF1A和RECK基因, miR-342的高表达可明显抑制裸鼠的肿瘤生长和肺转移。新发现的miR-342-DNMT1连系为CRC的治疗提供了潜在的治疗靶标^[10]^。

研究报道在多种人类癌症中存在不规则甲基化的miRNAs。一项研究显示在恶性胸膜间皮瘤(malignant pleural mesothelioma, MPM)细胞中可见异常甲基化的miR-34b/c, 在甲基化细胞中miR-34b/c的表达降低。强迫miR-34b/c的过表达具有抗增殖作用, 使发生G1期细胞周期停滞, 并可抑制转移、侵袭和活跃性。5-杂氮-2'-脱氧胞苷治疗后miR-34b/c的表达可恢复, 这提示miR-34b/c可作为MPM的潜在治疗选择方案^[11]^。另一项研究利用miRNA分析发现在肝细胞癌(hepatocellular carcinoma, HCC)细胞中miR-125b表达下降, 并且甲基化抑制剂5-杂氮-2'-脱氧胞苷也可增加miR-125b的表达。加入miR-125b前体可降低癌细胞增殖、非停泊性生长、细胞转移、侵袭及血管新生。研究发现PIGF可作为miR-125b的靶标, miR- 125b的过表达可降低HCC细胞中PIGF的表达并改变血管新生指数^[12]^。

### 2.2 miRNAs在核心信号通路中的潜在临床意义

近年来一些与肿瘤启动和进展有关的复杂肿瘤信号通路已被阐明。信号通路的深入了解驱动着靶向作用于特定分子事件的新一代抗癌治疗的发展^[13]^。在过去数年内已涌现出一些潜在的miRNA治疗药物, 用于抑制不同信号通路中的单个或多个靶点。

有研究报道在转移性乳腺癌细胞中miR-10b的表达增高, 另有研究发现通过TWIST1表达的抑制, CCN5可抑制miR-10b的表达, 该抑制由HIF-1α的转化抑制与修饰通过阻碍JNK信号通路而介导。miR-10b阳性转移性乳腺癌中CCN5的重新激活或可为现有的乳腺癌治疗提供又一候选策略^[14]^。一项在HCC中对miRNAs进行的深度分析发现HCC中miR-199a/b-3p表达降低。该miRNA可通过抑制PAK4/Raf/MEK/ERK通路靶向作用于促癌性PAK4而抑制在体和离体中HCC的生长, 他们的研究提示miR-199a/ b-3p可作为HCC的治疗靶标^[15]^。

雄激素受体(androgen receptor, AR)在前列腺癌变中起重要作用。有研究显示miR-488^*^的过表达可下调AR的转录活性并抑制外源性AR的蛋白生成。miR-488^*^可阻断PCa细胞的增殖并促进其凋亡。研究数据显示miR-488^*^不单可靶向作用于AR, 更为AR介导信号的潜在调节剂, 其在PCa中可作为靶向作用于AR的新疗法^[16]^。另一方面, 一些miRNAs在细胞增殖和凋亡中发挥作用。一项研究发现在乳腺癌细胞中miR-24-2通过调节不同的凋亡通路并靶向作用于BCL-2而诱导凋亡。过表达miR-24-2的细胞对抗癌药物高度敏感, miR-24-2与抗癌药物如顺铂的联合治疗或可为耐药肿瘤患者的抗癌治疗开辟一条新的道路^[17]^。

### 2.3 转移相关miRNAs为基础的治疗方法

新近证据显示miRNAs在肿瘤转移中发挥至为重要的作用。一项有关CRC的研究关注miR-499-5p作为候选的促转移miRNA并发现在高侵袭性CRC细胞中miR-499-5p水平升高。miR-499-5p表达增加可促进离体CRC细胞的转移和侵袭, 并促进在体的肺和肝转移, 而沉默其表达则可降低转移和侵袭。该研究已鉴定FOXO4和PDCD4为miR- 499-5p的直接功能性靶标, 这些结果提示miR-499-5p可促进CRC细胞的转移并可作为CRC的潜在治疗靶标^[18]^。

在侵袭性乳腺癌细胞中外源性miR-340的表达下调, 有研究显示增加miR-340的表达可抑制肿瘤细胞的转移和侵袭, 而敲除miR-340的表达则可诱导乳腺癌细胞转移和侵袭。研究发现通过调节MMP-2和MMP-9的表达, c-Met作为直接的miR-340靶标可介导细胞转移和侵袭, 这提示miR-340可作为乳腺癌转移的潜在治疗靶标^[19]^。另有研究显示miR-520b亦可抑制高转移性乳腺癌细胞的转移, HBXIP刺激NF-қB介导的IL-8表达促进癌细胞的转移网络, 通过靶向作用于HBXIP和IL-8, miR-520b可调节乳腺癌细胞的转移, 这些发现提示HBXIP可作为转移性乳腺癌的潜在治疗靶标^[20]^。

一项有关胃癌(gastric cancer, GC)的研究显示miR- 148a可发挥抑制肿瘤转移的作用, 过表达的miR-148a可抑制离体GC细胞的转移和侵袭以及在体肺转移灶的形成, 还发现ROCK1在miR-148a诱导的GC细胞转移和侵袭中发挥作用, 他们的结果提示miR-148a或具有抑制GC转移的治疗潜能^[21]^。也有研究关注miR-516a-3p作为GC的候选抗转移miRNA, 发现在高转移性细胞中miR-516a-3p的表达减弱, 并鉴定sulfatase 1为该miRNA的直接靶标。通过端胶原将miR-516a-3p表达载体传送至原位高转移性肿瘤中, 揭示了该miRNA用作治疗药物的可行性。这些结果显示miR-516-3p是一个抗转移miRNA, 在阻断GCs转移扩散中具有潜在的治疗价值^[22]^。

上皮-间质转化(epithelial-mesenchymal transition, EMT)在肿瘤侵袭和转移中发挥重要作用。有研究报道在人胰腺癌细胞中siRNA介导的DCAMKL-1敲除可诱导miR-200a并下调EMT相关转录因子ZEB1、ZEB2、Snail、Slug和Twist, 这些结果显示在胰腺癌中通过miR-200a依赖性机制DCAMKL-1作为调节EMT的治疗靶标是合理的^[23]^。另一方面, 化疗也可在肿瘤细胞中诱导EMT。一项研究发现在对化疗耐药的舌癌细胞中miR-15b和miR-200b均呈下调, 这些miRNAs的高表达可有效逆转EMT的表型并增强癌细胞的化疗敏感性, 而增加miR-15b或miR-200b的表达亦可抑制舌癌异种移植动物的癌转移。研究发现BMI1是miR-15b和miR-200b的靶标, 在舌癌中这些miRNAs或可作为治疗靶标逆转化疗的耐药性^[24]^。

## 3 专家点评

深入了解miRNAs的潜在分子机制将有助于把miRNAs转化应用为癌症的治疗靶标。miRNA治疗的一大优势为其可靶向作用于不同信号通路的多个基因。然而, 它也伴随有许多未知的脱靶效应的缺陷。尽管临床前肿瘤模型在体试验支持miRNAs为有效的抗癌药物^[25]^, 一些细微的生理变化(如肝脏和脾脏的大小变化、血清蛋白质及代谢物的浓度变化)表明对在体miRNA治疗而言, 非毒性转运的挑战仍然存在^[26]^。

在技术方面, 尽管miRNA易于合成, 但克服系统性转运的障碍对于有效的miRNA治疗来说仍是主要的难题。药物转运系统的快速发展为成功的miRNA治疗的出现展示了曙光, 一项新近临床前研究发现在小鼠非小细胞肺癌模型中, 化学合成的miR-34a和脂质传递载体构成的治疗配方可阻断肿瘤生长。该配方耐受性好, 且不诱导免疫反应, 此方法或可为miRNA替代治疗用于临床开辟一条快捷通道^[27]^。

临床前研究丰富了我们对癌症生物学中miRNAs功能性作用的了解, 这或可开发新的癌症治疗方法^[28]^。尽管有效且安全的miRNA癌症治疗手段的到来仍较为遥远, 新近研究的发现为该领域的发展提供了乐观的前景。
